# Breastfeeding in term and preterm infants with and without growth restriction: A 50‐year analysis of incidence and duration

**DOI:** 10.1002/ijgo.70770

**Published:** 2026-01-23

**Authors:** Achim Fieß, Alica Hartmann, Eva Mildenberger, Julia Winter, Mareike Ernst, Jonas Tesarz, Michael S. Urschitz, Norbert Pfeiffer, Alexander K. Schuster, Sandra Gißler, Dirk Wackernagel

**Affiliations:** ^1^ Department of Ophthalmology University Medical Center of the Johannes Gutenberg University Mainz Mainz Germany; ^2^ Division of Neonatology, Department of Pediatrics University Medical Center of the Johannes Gutenberg University Mainz Mainz Germany; ^3^ Department of Psychosomatic Medicine and Psychotherapy University Medical Center of the Johannes Gutenberg University Mainz Mainz Germany; ^4^ Department of Clinical Psychology, Psychotherapy and Psychoanalysis, Institute of Psychology University of Klagenfurt Klagenfurt am Woerthersee Austria; ^5^ Division of Pediatric Epidemiology, Institute of Medical Biostatistics, Epidemiology, and Informatics University Medical Center of the Johannes Gutenberg University Mainz Mainz Germany

**Keywords:** breastfeeding, fetal growth restriction, gestational age, maternal education, prematurity

## Abstract

**Objective:**

The aim of the present study was to analyze breastfeeding rates and duration in relation to preterm birth, fetal growth restriction, and demographics over five decades.

**Methods:**

This retrospective cohort study included 1559 individuals (aged 4–52 years) and their parents from the University Medical Center Mainz, Germany. Participants were categorized by gestational age (extremely preterm ≤28 weeks, very preterm 29–32 weeks, moderately preterm 33–36 weeks, term ≥37 weeks), and birth weight percentile (small for gestational age [SGA] <10th, appropriate for gestational age [AGA] 10th–90th, large for gestational age [LGA] >90th). Data were collected via interviews, questionnaires, and medical records.

**Results:**

Data from 940 mothers were analyzed. Breastfeeding rates and duration significantly increased over 50 years. However, low gestational age, particularly ≤28 weeks and 29–32 weeks and being born SGA were significantly associated with reduced breastfeeding incidence. Delayed breastfeeding initiation was more frequent in preterm and SGA‐born infants. Additionally, there was a significant positive association between year of birth and breastfeeding rates and duration. Higher maternal educational level correlated positively with breastfeeding initiation and duration.

**Conclusion:**

Breastfeeding rates and duration significantly increased over the past 50 years. However, both preterm birth and fetal growth restriction were associated with lower breastfeeding incidence and delayed initiation. Notably, being born SGA was independently linked to reduced breastfeeding, even after accounting for gestational age. While the association between prematurity and breastfeeding challenges is well established, our findings suggest that being born SGA may represent a similarly important but less widely recognized risk factor. This underlines the need for increased awareness and tailored breastfeeding support for this specific group.

**Abbreviations**
AGA: appropriate for gestational age
LGA: large for gestational age
NICU: neonatal intensive care unit
SGA: small for gestational age

## 1 INTRODUCTION

Preterm infants face a variety of medical challenges, including a higher susceptibility to infections,^1^ respiratory problems^2^ and altered long‐term developmental outcomes.^3^ Similarly, infants who are born small for gestational age (SGA) are at increased risk for long‐term complications such as non‐communicable diseases.^4^ These specific health challenges highlight the critical importance of postnatal care for such infants to reduce the risk of future health problems. In this context, breastfeeding plays a key role in supporting the long‐term health and development of these infants. Beyond the nutritional and immunological benefits of breast milk, breastfeeding also promotes emotional bonding between mother and child essential for the child's psychological development and emotional well‐being.^5^ Increased periods of separation of mother and infant limit the opportunities for bonding and interaction, thereby exacerbating the emotional distress experienced by mothers.^6^

Breast milk contains essential nutrients and bioactive components,^7^ playing a key role in supporting immune system development to protect against infections and inflammatory processes.^8^ Breast milk nutrition is also associated with a reduced risk of inflammatory diseases such as asthma and type 2 diabetes.^9^, ^10^ In the context of premature birth or low birth weight (BW), breastfeeding and the supply of breast milk are crucial in mitigating negative health outcomes, promoting postnatal growth^11^ and neurodevelopmental outcomes,^12^, ^13^ helping infants born preterm to achieve critical developmental milestones. Breast milk nutrition also reduces the risk of serious conditions like necrotizing enterocolitis in preterm infants.^14^

However, some parents and children face unique obstacles to breastfeeding. Preterm infants may not suckle efficiently at their mother's breast, making physiological lactation difficult,^15^, ^16^ thus, lactation initiation and advancement must be actively supported by frequent and circadian breast milk pumping. Additionally, prematurity‐related complications pose significant challenges accompanied by maternal anxiety and depression regarding the infant's health condition.^11^ Typically, preterm infants have a significantly shorter duration of breastfeeding,^17^, ^18^ not meeting the recommendation of the WHO of exclusive breastfeeding during the first six months.^19^ Over the past decades, global and national breastfeeding orientations and policies have changed substantially. Following the decline in breastfeeding during the mid‐20th century, initiatives such as the WHO/UNICEF Baby‐Friendly Hospital Initiative (BFHI; launched in 1991) promoted early skin‐to‐skin contact, rooming‐in and exclusive breastfeeding as standard hospital practices.^20^ The implementation and scaling‐up of BFHI and the related Baby‐Friendly Community Initiative (BFCI) have since been adopted in more than 150 countries, contributing to improved breastfeeding support structures, standardized hospital policies and increasing parental involvement in neonatal care units.^21^ Globally, the prevalence of exclusive breastfeeding for infants under six months of age remains highly heterogeneous. Across low‐ and middle‐income countries, the weighted prevalence of exclusive breastfeeding under six months was 45.7% during 2010–2018, with substantial regional variation ranging from 34.5% in the Eastern Mediterranean region and 43.7% in European regions to 55.2% in South‐East Asia and the Western Pacific region.^22^

Breastfeeding rates have changed significantly but trends in premature or fetal growth‐restricted populations remain poorly understood.^23^ Although the impact of prematurity on breastfeeding rates has been investigated,^24^, ^25^ there is often insufficient differentiation between different grades of prematurity and fetal growth restriction, leading to an incomplete understanding of their potentially distinct effects on breastfeeding and the unique challenges faced by mothers. Furthermore, there is a lack of long‐term data describing long‐term changes in breastfeeding rates despite significant advancements in perinatal medicine during this time. The aim of the present study was to address this gap by examining breastfeeding rates and duration over a 50‐year period in a German University Hospital considering the degrees of prematurity and fetal growth restriction.

## 2 MATERIALS AND METHODS

### 2.1 Study population

**FIGURE 1:**
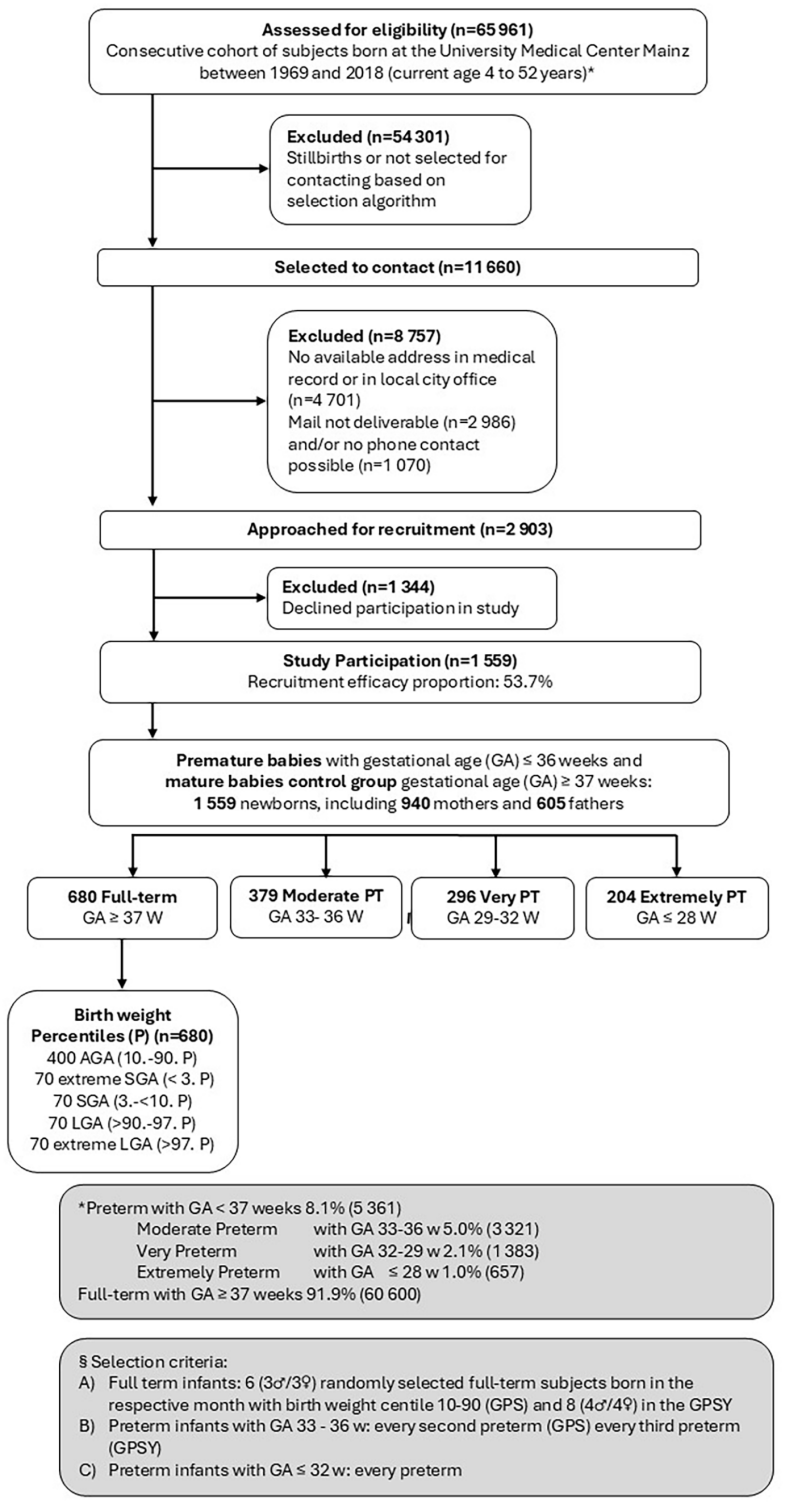
Study design of the Gutenberg Prematurity Study.

The Gutenberg Prematurity Study is a retrospective cohort study with prospective examinations and parental interviews conducted at the University Medical Center Mainz (UMCM), Germany. It includes participants born preterm or at term between 1969 and 2018 and aged between 4 and 52 years old at the time of enrolment. Every second individual born preterm with a gestational age of 33 to 36 weeks from 1969 onward, as well as all individuals born preterm at or before 32 weeks of gestation at UMCM, were invited to participate. Six term‐born individuals (3 males and 3 females) with birth weights between the 10th and 90th percentiles were selected from every month between 1969 and 2018 to form an age‐ and sex‐matched control group. Also, 140 term individuals born SGA (BW below the 10th percentile) and 140 large for gestational age (LGA, above the 90th percentile) were recruited to study the effects of escalating degrees of fetal growth independent of prematurity. All participants were matched by age and sex at examination. The inclusion and exclusion process of participants is visible in Figure 1, while the age distribution is presented in Figure S1.

Participants were stratified by gestational age: Group 1 included 680 participants born at 37^+0^ weeks of gestation or later; Group 2 comprised 379 participants born between 33^+0^ and 36^+6^ weeks; Group 3 consisted of 296 participants born between 29^+0^ and 32^+6^ weeks; Group 4 included 204 participants born before 29^+0^ weeks. They underwent detailed medical evaluations and interviews between 2019 and 2023. The parents were also invited to complete a questionnaire, resulting in 419 responses from mothers in Group 1, 231 in Group 2, 169 in Group 3, and 121 in Group 4.

All participants provided written informed consent before joining the study following the standards of Good Clinical Practice, Good Epidemiological Practice, and the ethical guidelines of the Declaration of Helsinki. The study's protocol and related documents were approved by the local ethics committee (reference number anonymized).

### 2.2 Data collection

#### 2.2.1 Breastfeeding rate, initiation, and duration

Initially, all participants and their participating parents completed a sociodemographic questionnaire. Data from mothers were primarily obtained through standardized written questionnaires. Only a small proportion of parents participated in follow‐up interviews, as questionnaire data could be validated directly with medical records for most participants. For participants whose parents were not available for interview, information from the standardized parental questionnaire together with verified medical records from birth and the neonatal period was used. All interviews followed a structured protocol, and responses were cross‐checked with medical record entries whenever possible to ensure data reliability. Participating mothers were asked whether they had breastfed their baby after birth (yes/no). If they answered “yes”, they were further asked when they first breastfed their baby (response options: on the first day, during the first week after day 1, or in the second week) and for how long.

To account for variation in neonatal maturity, especially among preterm infants, we documented the timing of breastfeeding initiation rather than using a uniform cutoff for “successful breastfeeding.” Given that extremely and very preterm infants are physiologically often unable to feed orally in the first days of life, delayed initiation (>1 day postpartum) was not interpreted as unsuccessful per se, but as a marker to reflect clinical realities. Therefore, our analysis focused on breastfeeding incidence, timing of initiation, and duration rather than using a rigid definition of “successful breastfeeding.”

More detailed questions regarding in‐hospital nutrition were also evaluated, including whether the infant was exclusively fed with mother's milk, formula, a combination of both or donor milk. The same questions were asked about nutrition after discharge. Finally, parents were asked whether they experienced any feeding difficulties after discharge (yes/no) and for how long in months.

#### 2.2.2 Pre‐ and postnatal history

Information regarding gestational age in weeks, BW in kilograms, multiple births, placental insufficiency, maternal hemolysis, elevated liver enzymes, low platelet count (HELLP) syndrome, pre‐eclampsia, maternal smoking, maternal alcohol consumption, perinatal adverse events, necrotizing enterocolitis, moderate/severe bronchopulmonary dysplasia, intubation and duration of stay in the neonatal intensive care unit (NICU, days) were obtained from medical chart reviews and maternal interviews. BW percentiles were calculated using growth charts by Voigt et al.^26^ Perinatal adverse events were defined based on the German quality assessment guidelines for neonatal units including intraventricular hemorrhage (grade 3 or parenchymal hemorrhage), necrotizing enterocolitis, and/or moderate to severe bronchopulmonary dysplasia.^27^ The mode of enteral feeding was assessed using medical records and categorized as exclusively mother's milk, formula, a combination of both or donor milk.

### 2.3 Statistical analysis

Multivariable logistic regression was applied to analyze the primary outcomes reported by the mothers: breastfeeding (yes), delayed first breastfeeding (>1 day), total breastfeeding duration (<6 months), and feeding problems after discharge (yes). The regression models included the following independent variables: model 1 included gestational age group categories (≤28 weeks, 29–32 weeks, and 33–36 weeks) (reference category: ≥37 weeks), age of mother at delivery, year of birth, multiple births (yes) and mother's education (categorized into “high” for higher level secondary education and “lower” for basic intermediate school); model 2 included gestational age groups, the mother's age at delivery, year of birth, multiple births (yes), mother's education and BW percentile groups (SGA and LGA). The gestational age group age of ≥37 weeks and appropriate for gestational age (AGA) (BW percentile 10–90) was used as a reference. Based on the WHO recommendations, the cutoff for the total breastfeeding duration was set to 6 months^19^ to distinguish between the mother falling short of or extending the recommended minimum breastfeeding duration by the WHO. A sensitivity analysis was performed using gestational age and BW percentile as continuous variables to increase statistical power. All statistical analyses were conducted using R version 4.3.2.

## 3 RESULTS

The study included 1559 individuals (median age 15 years, range: 4–52 years; 824 females), and the participant characteristics are presented in Table 1 stratified by study group. The age distribution at the study examination is shown in Figure S1. Notably, breastfeeding rates decreased with gestational age, with the lowest rate in the gestational age ≤ 28 weeks group (*n* = 103/204, 50%). After hospital discharge, the preterm groups were fed more often on formula or a combination of formula and breast milk compared to term infants. Feeding problems also tended to last longer in the preterm groups, with the longest duration observed in the gestational age ≤ 28 weeks group (mean: 8.50 months, standard deviation [SD]: 7.84 months; Table 2).

**TABLE 1:** Characteristics of the participants (*n* = 1559) stratified by gestational age group.

	Group 1	Group 2	Group 3	Group 4
	GA ≥37 weeks	GA 33–36 weeks	GA 29–32 weeks	GA ≤28 weeks
Number of participants (*n*)	680	379	296	204
Age at examination (years), median [min, max]	16 [4, 51]	15 [4, 51]	16 [4, 52]	14 [4, 45]
Women	354 (52.1%)	202 (53.3%)	155 (52.4%)	113 (55.4%)
Birth weight, mean ± SD	3407 ± 765	2238 ± 449	1536 ± 365	827 ± 241
Birth weight percentile, mean ± SD	47.68 ± 35.03	30.83 ± 23.66	41.17 ± 24.31	37.62 ± 26.05
SGA (BW percentile <10)	140 (20.6%)	88 (23.2%)	28 (9.5%)	39 (19.1%)
AGA (BW percentile 10–90)	400 (58.8%)	285 (75.2%)	266 (89.9%)	163 (79.9%)
LGA (BW percentile >90)	140 (20.6%)	6 (1.6%)	2 (0.7%)	2 (1.0%)
Gestational age (weeks), mean ± SD	39.09 ± 1.39	34.52 ± 1.02	30.70 ± 1.11	25.89 ± 1.60
Range (min‐max)	(37–43)	(33–36)	(29–32)	(23–28)
Maternal age at study interview	50.1 ± 9.9	47.9 ± 9.2	49.0 ± 8.6	46.8 ± 9.2
Maternal age at childbirth	32.1 ± 4.7	33.6 ± 5.0	31.8 ± 5.0	32.5 ± 4.8
Multiple births (yes), *n* (%)	57 (8.4%)	157 (41.4%)	125 (42.2%)	62 (30.4%)
Preeclampsia (yes), *n* (%)	39 (5.7%)	45 (11.9%)	49 (16.6%)	34 (16.7%)
Placental insufficiency (yes), *n* (%)	15 (2.2%)	24 (6.3%)	9 (3.0%)	14 (6.9%)
HELLP syndrome (yes), *n* (%)	1 (0.1%)	14 (3.7%)	23 (7.8%)	11 (5.4%)
Gestational diabetes (yes), *n* (%)	51 (7.5%)	26 (6.9%)	23 (7.8%)	8 (3.9%)
Perinatal adverse events (yes), *n* (%)	1 (0.1%)	5 (1.3%)	20 (6.8%)	100 (49.0%)
Necrotizing enterocolitis, *n* (%)	0 (0.0%)	4 (1.1%)	8 (2.7%)	20 (9.8%)
Moderate/severe bronchopulmonary dysplasia, *n* (%)	1 (0.1%)	1 (0.3%)	13 (4.4%)	83 (40.7%)
Intubation, *n* (%)	6 (0.9%)	33 (8.7%)	109 (36.8%)	179 (87.7%)
Length of stay in the NICU (days), median (IQR)	0.00 [0.00, 0.00]	0.00 [0.00, 4.00]	15.00 [6.00, 28.00]	65.00 [46.00, 89.00]
Maternal smoking during pregnancy (yes), *n* (%)	30 (4.4%)	15 (4.0%)	18 (6.1%)	23 (11.3%)
Maternal alcohol consumption during pregnancy (yes), *n* (%)	7 (1.0%)	2 (0.5%)	4 (1.4%)	6 (2.9%)

*Note*: Appropriate‐for‐gestational‐age [AGA] 10th–90th, large‐for‐gestational‐age [LGA].

Abbreviations: AGA, appropriate for gestational age; BW, birth weight; GA, gestational age; HELLP syndrome, hemolysis, elevated liver enzymes, and low platelet count syndrome; IQR, interquartile range; LGA, large for gestational age; NICU, neonatal intensive care unit; SD, standard deviation; SGA, small‐for‐gestational age.

**TABLE 2:** Breastfeeding, neonatal, and post‐discharge nutrition stratified by gestational age group.

	Group 1	Group 2	Group 3	Group 4	*P* value
	GA ≥37 weeks	GA 33–36 weeks	GA 29–32 weeks	GA ≤28 weeks	
Number of participants	680	379	296	204	
Breastfeeding					
Breastfed after birth (yes), *n* (%)	494 (72.6%)	258 (68.1%)	183 (61.8%)	103 (50.5%)	<0.001
Total duration of breastfeeding (months), mean ± SD	7.09 ± 5.96	5.78 ± 5.07	6.16 ± 5.93	5.29 ± 6.03	0.28
Nutrition during hospital stay					
Number of responses regarding nutrition	379	203	151	103	
Exclusively mother's milk (yes), *n* (%)	258 (68.1%)	93 (45.8%)	62 (41.1%)	59 (57.3%)	<0.001
Exclusively formula (yes), *n* (%)	58 (16.6%)	50 (24.6%)	35 (23.2%)	20 (19.4%)	0.07
Breastmilk and formula (yes), *n* (%)	63 (15.3%)	60 (29.6%)	54 (35.8%)	24 (23.3%)	<0.001
Donor milk (yes), *n* (%)	1 (0.25%)	5 (2.3%)	9 (5.4%)	1 (0.9%)	<0.001
Nutrition after discharge					
Number of responses regarding nutrition	386	206	155	105	
Exclusively mother's milk (yes), *n* (%)	274 (71.0%)	109 (52.9%)	69 (44.5%)	45 (42.9%)	<0.001
Exclusively formula (yes), *n* (%)	66 (17.1%)	64 (31.1%)	57 (36.8%)	39 (37.1%)	<0.001
Breastmilk and formula (yes), *n* (%)	46 (11.9%)	33 (16.0%)	29 (18.7%)	21 (20%)	0.002
Feeding problems after discharge (yes), *n* (%)	66 (17.6%)	42 (21.0%)	21 (14.0%)	24 (23.5%)	0.14
Duration of feeding problems (months), mean ± SD	4.65 ± 6.69	6.72 ± 12.81	5.86 ± 5.40	8.50 ± 7.84	0.34

Abbreviations: BW, birth weight; GA, gestational age; SD, standard deviation.

Stratification by breastfeeding group (no breastfeeding, immediate breastfeeding, and delayed breastfeeding) revealed that individuals who were not breastfed or experienced delayed breastfeeding were born at a significantly lower gestational age and with a lower BW percentile. These groups also had a higher prevalence of perinatal adverse events compared to the immediate breastfeeding group with higher rates of necrotizing enterocolitis, bronchopulmonary dysplasia, use of intubation, and longer NICU stays in the infants who were not breastfed. Furthermore, type 2 diabetes was more frequently reported in adults who had not been breastfed and not observed in the other two groups. Additionally, epilepsy and hypertension were significantly more common in the no‐breastfeeding and delayed breastfeeding groups compared to the immediate breastfeeding group (Table S1).

### 3.1 Breastfeeding rate and infants' nutrition (over 50 years)

**FIGURE 2:**
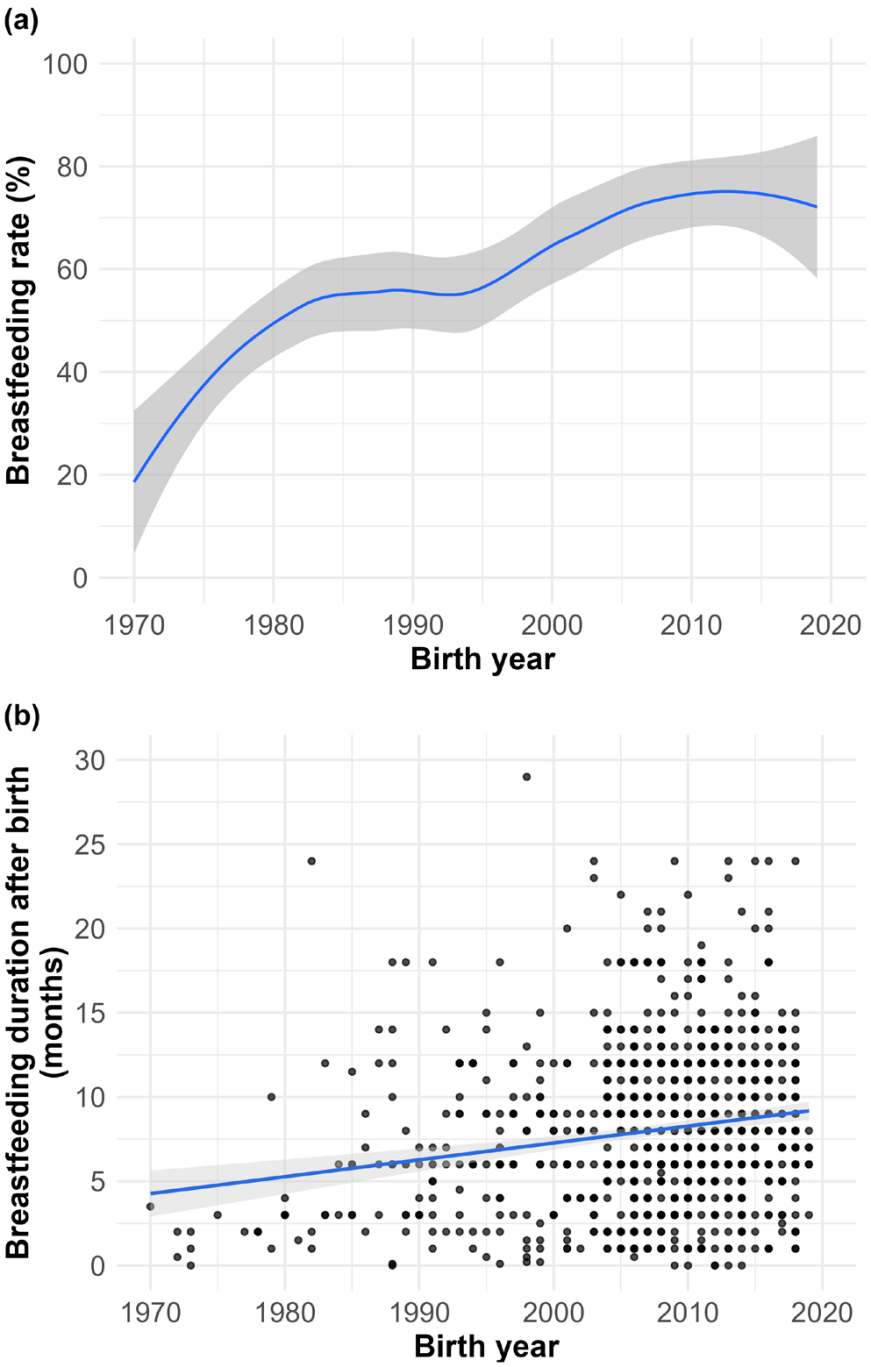
Breastfeeding rates (a) and breastfeeding duration in months (b) over five decades.

**FIGURE 3:**
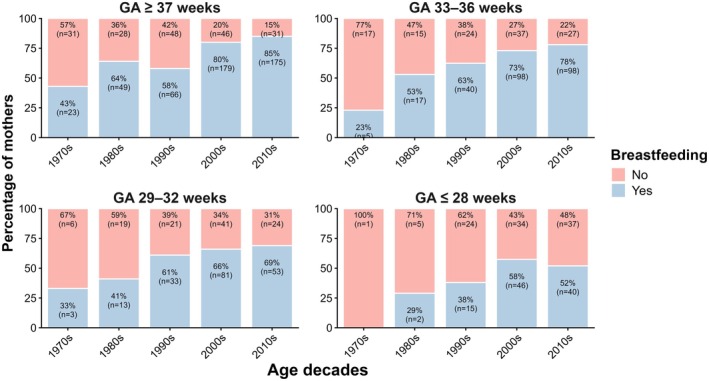
Percentage of mothers who breastfed their baby (yes/no) stratified by gestational age (GA) groups over five decades.

**FIGURE 4:**
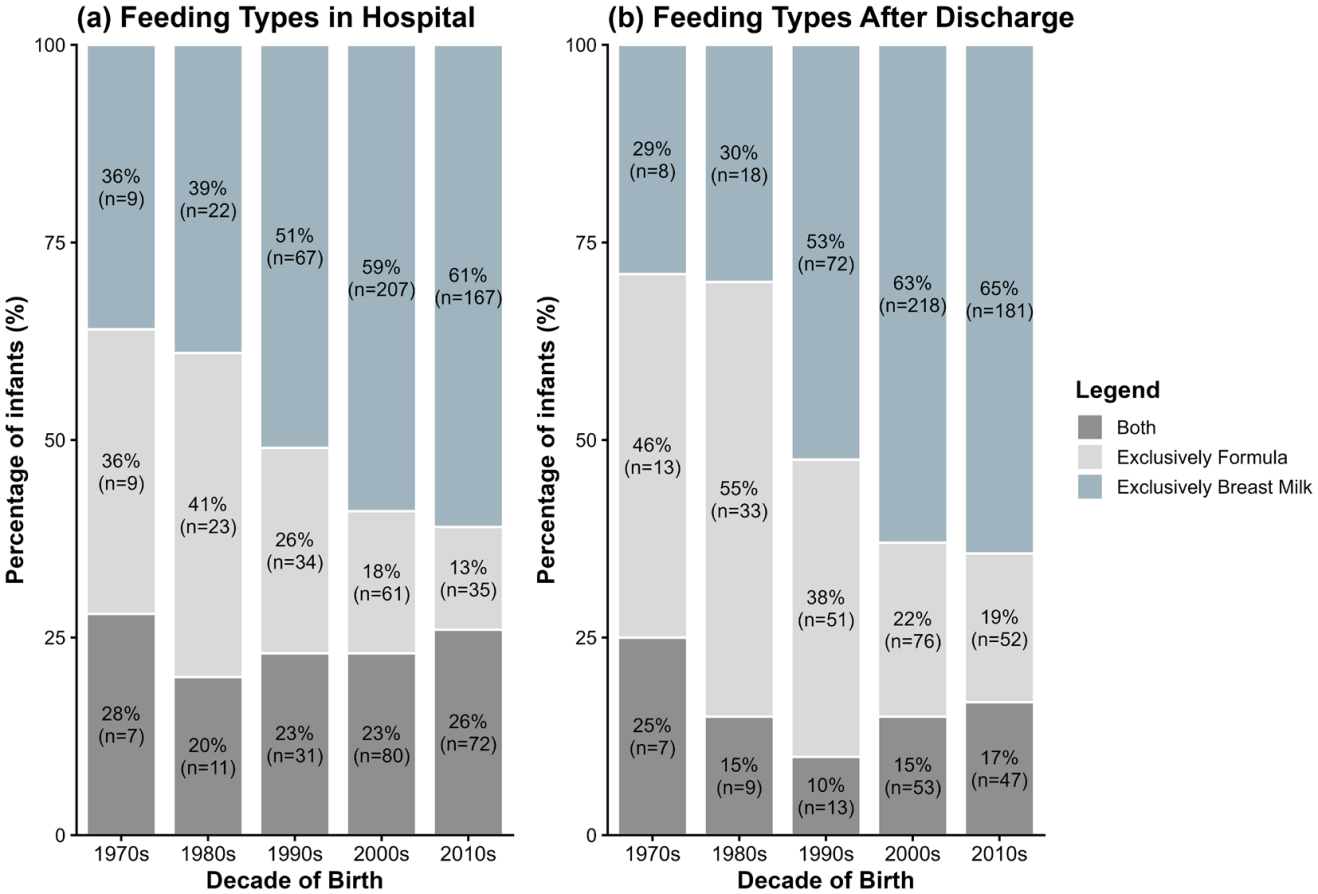
Trends in infant feeding types: In‐hospital and after discharge over five decades.

Figure 2 and Figure 3 illustrate the overall increase in breastfeeding rates and duration over time in both preterm (all degrees) and term infants. In the hospital, the proportion of infants exclusively fed breast milk increased steadily over the decades, accompanied by a marked decline in formula feeding, while the mix of both remained relatively stable (Figure 4a). After discharge, a similar upward trend in exclusive breastfeeding was observed, with declines in both exclusive formula feeding and mixed feeding (Figure 4b).

### 3.2 Factors associated with breastfeeding rates after birth

The multivariable logistic regression analysis revealed that both extremely and moderately preterm infants had significantly reduced odds of being breastfed, with a higher maternal educational status and more recent birth year associated with an increased likelihood of breastfeeding. Fetal growth restriction was associated with lower odds of being breastfed (Table 3). Delayed initiation of breastfeeding was observed across all preterm groups and SGA infants also showed delayed breastfeeding initiation. Notably, the likelihood of delayed initiation decreased significantly with a more recent birth year. Mothers with multiple births were more likely to experience delayed breastfeeding initiation and shorter duration of breastfeeding. Moreover, more recent years of birth were associated with longer breastfeeding duration, and feeding difficulties were more frequently observed in infants born SGA and among mothers with a higher educational status, whereas feeding difficulties were less common among older mothers (Table 4).

**TABLE 3:** Association analyses breastfeeding rates after birth of infants born preterm and full‐term (*N* = 1559).

	Model 1		Model 2: With additional integration of birth weight percentile groups	
	OR (95% CI)	*P* value	OR (95% CI)	*P* value
Breastfeeding (yes)				
GA ≤28 weeks	0.32 (0.22, 0.47)	<0.001	0.33 (0.23, 0.49)	<0.001
GA 29–32 weeks	0.65 (0.46, 0.90)	<0.001	0.63 (0.45, 0.90)	0.01
GA 33–36 weeks	0.88 (0.64, 1.22)	0.45	0.93 (0.67, 1.29)	0.65
Age of mother at birth	0.99 (0.98, 1.01)	0.31	1.00 (0.97, 1.00)	0.27
Year of birth	1.04 (1.03, 1.06)	<0.001	1.04 (1.03, 1.06)	<0.001
Mother's education (high)^a^	1.66 (1.30, 2.12)	<0.001	1.60 (1.25, 2.05)	<0.001
Multiple births (yes)	0.73 (0.55, 0.97)	0.03	0.77 (0.58, 1.02)	0.07
SGA <10 BW percentile			0.63 (0.47, 0.85)	0.002
LGA >90 BW percentile			1.37 (0.87, 2.19)	0.19

Abbreviations: BW, birth weight; CI, confidence interval; GA, gestational age; LGA, large for gestational age; OR, odds ratio; SGA, small for gestational age.

^a^ Mother's school‐leaving qualification (high) was defined as “high” for higher secondary school completion, such as “gymnasium” or university degree, and “lower” for basic “Hauptschule” or intermediate school “Realschule”; a gestational age of ≥37 weeks was used as the reference category for the gestational age groups.

**TABLE 4:** Subgroup analyses of breastfeeding initiation, duration, and feeding problems in infants born preterm and term for breastfed participants (breastfeeding = yes) (*n* = 1038).

	Model 1		Model 2: With additional integration of birth weight percentile groups	
	OR (95% CI)	*P* value	OR (95% CI)	*P* value
Delayed initiation of breastfeeding (>1 day)				
GA ≤28 weeks	2.61 (1.36, 4.88)	<0.001	2.40 (1.25, 4.53)	<0.001
GA 29–32 weeks	6.44 (1.36, 10.71)	<0.001	6.27 (3.75, 10.62)	<0.001
GA 33–36 weeks	4.84 (3.12, 7.59)	<0.001	4.57 (2.90, 7.27)	<0.001
Age of mother at birth	1.05 (0.99, 1.11)	0.08	1.06 (1.00, 1.11)	0.05
Year of birth	0.97 (0.95, 1.00)	0.04	0.97 (0.95, 1.00)	0.02
Mother's education (high)	0.75 (0.44, 1.27)	0.28	0.80 (0.47, 1.37)	0.42
Multiple births (yes)	2.93 (1.48, 5.98)	0.002	2.27 (1.13, 4.70)	0.02
SGA <10 BW percentile			2.40 (1.22, 4.77)	0.02
LGA >90 BW percentile			0.71 (0.30, 1.54)	0.41
Total breastfeeding duration (<6 months)				
GA ≤28 weeks	0.89 (0.50, 1.53)	0.67	0.85 (0.48, 1.48)	0.58
GA 29–32 weeks	1.12 (0.71, 1.75)	0.63	1.06 (0.66, 1.69)	0.80
GA 33–36 weeks	1.39 (0.94, 2.04)	0.10	1.33 (0.89, 1.98)	0.16
Age of mother at birth	0.96 (0.93, 1.00)	0.04	0.97 (0.93, 0.99)	0.04
Year of birth	0.96 (0.95, 0.98)	<0.001	0.96 (0.94, 0.97)	<0.001
Mother's education (high)	0.64 (0.47, 0.89)	0.01	0.64 (0.47, 0.89)	0.01
Multiple births (yes)	1.74 (1.20, 2.54)	0.004	1.73 (1.18, 2.54)	0.004
SGA <10 BW percentile			0.96 (0.62, 1.47)	0.86
LGA >90 BW percentile			0.78 (0.44, 1.34)	0.38
Feeding problems after discharge (yes)				
GA ≤28 weeks	1.26 (0.59, 2.58)	0.53	1.15 (0.53, 2.39)	0.71
GA 29–32 weeks	0.39 (0.16, 0.87)	0.03	0.40 (0.16, 0.88)	0.03
GA 33–36 weeks	1.08 (0.63, 1.85)	0.77	0.97 (0.56, 1.68)	0.92
Age of mother	0.94 (0.89, 0.99)	0.02	0.94 (0.89, 0.99)	0.03
Year of birth	1.03 (1.00, 1.06)	0.05	1.03 (1.00, 1.05)	0.07
Mother's education (high)^a^	1.61 (0.96, 2.76)	0.08	1.75 (1.03, 3.05)	0.04
Multiple births (yes)	1.44 (0.78, 2.60)	0.23	1.22 (0.66, 2.21)	0.52
SGA <10 BW percentile			2.28 (1.31, 3.94)	0.003
LGA >90 BW percentile			0.49 (0.18, 1.16)	0.13

Abbreviations: BW, birth weight; CI, confidence interval; GA, gestational age; LGA, large for gestational age; OR, odds ratio; SGA, small for gestational age.

^a^ Mother's education (high) was defined as “high” for higher secondary school completion, such as “gymnasium” or university degree, and “lower” for basic “Hauptschule” or intermediate school “Realschule”; a gestational age of ≥37 weeks was used as the reference category for the gestational age groups.

The sensitivity analysis revealed a dose‐dependent effect of gestational age and BW, with each lower week of gestational age associated with a lower likelihood of breastfeeding. A similar dose‐dependent relationship between a lower BW percentile and a reduced likelihood of breastfeeding was observed. Additionally, this analysis confirmed the positive association between year of birth and breastfeeding frequency (Table S2). Moreover, weeks of prematurity were significantly associated with delayed initiation of breastfeeding with a lower BW percentile associated with shorter breastfeeding duration (Table S3).

## 4 DISCUSSION

This study investigated the complex relationships between preterm birth, breastfeeding rates, and duration and feeding difficulties. First, breastfeeding rates increased significantly over time in all preterm groups and term infants. Breastfeeding duration also showed an increasing trend during the same period. Second, our findings revealed that lower gestational age, particularly ≤28 weeks was significantly associated with lower odds of breastfeeding compared to term infants, and breastfeeding initiation was significantly delayed in all preterm groups. SGA at birth was associated with a lower likelihood of breastfeeding and delayed initiation of breastfeeding with gestational age and BW percentiles dose‐dependently associated with the breastfeeding rate.

### 4.1 Changes in the breastfeeding rate over 50 years

There was a significant increase in breastfeeding rates and duration in our institution over the last five decades. Notably, our study is the first to provide data on long‐term changes in breastfeeding rates, complemented by a detailed evaluation of medical records at birth. Data from the KiGGS study (German Health Interview and Examination Survey for Children and Adolescents) show an increased breastfeeding rate from 2001 to 2008, while breastfeeding duration did not change.^28^ A U.S. study analyzing breastfeeding rates from 1991 to 2002 reported a similar increasing trend, with a 9.8% increase explained by maternal age and 11.5% by maternal education.^23^ There was no association between maternal age and breastfeeding rates in our cohort, however, a higher educational status was significantly associated with higher breastfeeding rates in line with other studies demonstrating that education plays a critical role in breastfeeding rates. Van Rossem et al. reported that mothers with a higher educational status in a population‐based cohort (*n* = 2914) were 22.4 times more likely to breastfeed their infants.^29^ The rising breastfeeding rates over the past 50 years can in part be explained by the improvements in maternal education. Even after correcting for maternal education, the association between the breastfeeding rates and year of birth remained significant. In the mid‐20th century, formula companies heavily marketed their products as a more reliable and modern alternative to breast milk, significantly influencing societal perceptions and contributing to a decline in breastfeeding rates. Subsequently, educational and political initiatives reversed these trends,^30^ with prenatal breastfeeding education/teaching courses increasing breastfeeding initiation and duration. Mothers who participated in such courses were more likely to establish breastfeeding.^31^ Efforts at the political level have also contributed to increasing breastfeeding rates, particularly among groups with low socioeconomic status. In Germany, the “Becoming Breastfeeding Friendly (BBF)” project, a collaboration between the Yale School of Public Health and the Federal Ministry of Food and Agriculture, from 2017 to 2019 assessed the framework conditions for breastfeeding and led to recommendations such as the right to breastfeed in the workplace and discussed the establishment of standards for evidence‐based breastfeeding counseling.^32^

### 4.2 Factors associated with breastfeeding rate, initiation, and duration

Both preterm birth and fetal growth restriction were significantly associated with lower breastfeeding rates and delayed initiation of breastfeeding compared to term and appropriately grown infants. Notably, in our cohort, being born SGA was independently associated with reduced breastfeeding rates and later initiation, even after accounting for gestational age. This finding highlights the need to consider fetal growth restriction as a distinct risk factor, not only in the context of preterm birth. Crippa et al. analyzed late preterm infants (gestational age 34–36 weeks and BW ≥1800 g) emphasizing that a large proportion of these infants had breastfeeding difficulties and only a small proportion was exclusively breastfed.^33^ Maastrup et al. found that preterm infants with a gestational age of less than 32 weeks experienced delayed breastfeeding initiation and this delay was also observed in SGA infants who concomitantly initiated breastfeeding later.^25^ Hackman et al. reported breastfeeding rates after one month of 63.8% in preterm infants (gestational age 34–36 weeks) compared to a significantly higher rate in term or post‐term infants of 76.5%.^24^

An immature sucking reflex is common among extremely preterm infants^15^, ^16^ making breastfeeding requiring the coordination of sucking, swallowing, and breathing more challenging. An observational study of preterm infants examined the intraoral vacuum, tongue movement, and milk intake of babies during breastfeeding, showing that the intraoral vacuum forces are comparable to those of term infants at the beginning of breastfeeding but decrease over time as the infants become exhausted, particularly when nipple shields are used. However, there was no close association between the extent of the intraoral vacuum and milk intake, but the duration of active sucking was significantly positively associated with the milk intake.^34^ As premature infants suck more strongly at the breast and have more problems coordinating sucking, swallowing, and breathing, this can lead to faster exhaustion and shorter breastfeeding duration.^15^, ^16^ In addition, medical problems can complicate or obstruct the initiation of breastfeeding, such as respiratory instability^35^ or necrotizing enterocolitis^36^ and a general increase in fatigue can also lead to inefficient breastfeeding.^37^ Additionally, the medically challenging situation of premature infants often results in increased maternal stress^38^ with frequent separations from their infant minimizing bonding and interaction opportunities, increasing the mothers' emotional stress, and is negatively associated with initiation of lactation and milk volume.^39^, ^40^ Moreover, many mothers of premature infants are unable to initiate breastfeeding and are therefore dependent on pumping which can contribute to greater physical exhaustion and breast discomfort. Breastmilk‐pumping mothers may also experience an insufficient milk supply.^41^, ^42^ In SGA infants, the prevalence of medical complications such as hypoglycemia^43^ and thermoregulatory problems^44^ is increased and their reduced strength and faster exhaustion compared to AGA infants can delay the initiation and further complicate breastfeeding routines. In summary, the combination of different sucking patterns during breastfeeding^15^, ^16^ along with medical complications^35^ and maternal stress^38^ may contribute to lower breastfeeding rates and delayed breastfeeding initiation in individuals born preterm or SGA. However, interventions such as kangaroo care have been shown to mitigate some of these challenges. Over recent decades, kangaroo care has become a standard practice in NICUs and has been shown to enhance mother–infant bonding, promote physiological stability, and facilitate earlier and more successful initiation of breastfeeding in preterm infants.^45^, ^46^ The increasing adoption of this practice likely contributed to improved breastfeeding outcomes in our cohort.

The mothers in our cohort were asked whether they had any feeding difficulties after discharge from the hospital and there was a significant association between SGA and post‐discharge feeding difficulties, possibly accounting for SGA infants not achieving appropriate weight gain after discharge, as they often take longer to reach significant growth milestones.^39^, ^40^ This challenge to promote growth after birth could lead to uncertainty and stress for mothers as they strive to ensure their infant's weight gain.

The significant increase in breastfeeding rates and duration over the past five decades for infants born preterm and term reflects an important positive development in neonatal care, highlighting the success of health interventions and education programs to promote and support breastfeeding. However, there were significant differences between infants born preterm and term, particularly in breastfeeding rates and breastfeeding initiation. The observed lower likelihood of breastfeeding among preterm infants may be intricately linked to underlying socioeconomic factors and maternal stressors. As noted, the experience of preterm birth itself often entails significant emotional and psychological stress,^47^ compounded by the medical complexities associated with neonatal intensive care^48^ which can impact maternal–infant bonding and breastfeeding confidence.^49^ Additionally, socioeconomic challenges, such as limited access to lactation support,^50^ workplace inflexibility,^51^ and financial insecurity,^52^ can further impede breastfeeding initiation and continuation. These stressors may interact synergistically, as mothers experiencing preterm birth are often in a more vulnerable socioeconomic position^53^ and must navigate heightened demands on time and emotional resources, exacerbating barriers to breastfeeding. Addressing these challenges necessitates a holistic approach, including enhanced psychosocial support, targeted lactation counseling, and policies that alleviate structural inequities to promote breastfeeding in this vulnerable population. In addition to these psychosocial and structural measures, the implementation of standardized lactation support programs and early postnatal follow‐up visits can further promote breastfeeding success in preterm and SGA infants. Integrating lactation consultants into neonatal and follow‐up care, offering mother‐to‐mother support groups and ensuring continued education for healthcare professionals on breastfeeding practices have all been shown to improve breastfeeding initiation and duration.^54^, ^55^, ^56^ Furthermore, future studies should consider including maternal occupation as an additional socioeconomic factor, as work‐related stress, maternity leave duration, and occupational flexibility may substantially influence breastfeeding initiation and duration.^57^

Increased attention and support during breastfeeding is crucial for preterm infants as breastfeeding not only promotes bonding between mother and child,^58^, ^59^ but also plays an important role in supporting the immune system and long‐term health development.^60^, ^61^ Greater emphasis should also be placed on improving breastfeeding rates in infants born SGA to ensure that this vulnerable group can fully benefit from the protective and developmental advantages of breastfeeding.

This study had several limitations. First, the findings may not be fully generalizable as it was conducted at a single center with a hospital‐based cohort. There were challenges in reaching participants and some chose not to participate, potentially introducing selection bias. Additionally, most participants were of white ethnicity limiting the applicability of the conclusions to this demographic. The reliance on self‐report questionnaires and retrospective reporting by both mothers could introduce recall bias.

Despite these limitations, the study involved a large cohort of individuals born preterm and term with and without growth restriction over the past 50 years, with different degrees of prematurity and fetal growth, alongside a substantial control group. Detailed descriptions were provided by their mothers, and a thorough assessment of perinatal medical history was conducted, including a review of medical records, ensuring high validity of perinatal breastfeeding data during the hospital stay.

## 5 CONCLUSIONS

While breastfeeding rates and duration have significantly improved over the past 50 years, mothers of individuals born preterm and SGA continue to face specific challenges in initiating and maintaining breastfeeding. Our findings indicate that being born SGA is independently associated with reduced breastfeeding rates, highlighting its relevance as a risk factor alongside prematurity. Targeted support strategies are therefore essential to address these disparities and improve breastfeeding outcomes in these vulnerable groups.

## AUTHOR CONTRIBUTIONS

Alexander K. Schuster conceptualized and designed the study and contributed to validation and interpretation of the data. Achim Fieß conceptualized the study, codrafted the initial manuscript, and contributed to validation and interpretation of the data. Alica Hartmann codrafted the initial manuscript and conducted the initial analysis. Eva Mildenberger, Julia Winter, Mareike Ernst, Jonas Tesarz, Michael S. Urschitz, Norbert Pfeiffer, Sandra Gißler, and Dirk Wackernagel contributed to further validation, analysis, and interpretation of the data. All authors critically reviewed and revised the manuscript, approved the final version, and agree to be accountable for all aspects of the work.

## FUNDING INFORMATION

The Gutenberg Prematurity Study was supported by the Ernst und Berta‐Grimmke Stiftung, Stufe 1 support of the UM and the Else Kröner‐Fresenius‐Stiftung. The funders had no role in study design, data collection and analysis, decision to publish, or preparation of the manuscript. Schuster AK holds the professorship for ophthalmic healthcare research endowed by “Stiftung Auge” and financed by “Deutsche Ophthalmologische Gesellschaft” and “Berufsverband der Augenärzte Deutschlands e.V.”.

## CONFLICT OF INTEREST STATEMENT

Norbert Pfeiffer receives financial support and grants from Novartis, Ivantis, Santen, Thea, Boehringer Ingelheim Deutschland GmbH & Co. KG, Alcon, and Sanoculis. Alexander K. Schuster receives research support from Allergan, Bayer, Heidelberg Engineering, PlusOptix and Novartis. The other authors have no relevant conflicts to disclose.

## Supporting information


Appendix S1:

## Data Availability

Data are available upon reasonable request. The analysis presents the clinical data of a cohort. This project constitutes a major scientific effort with high methodological standards and detailed guidelines for analysis and publication to ensure scientific analyses are on the highest level, therefore, data are not made available for the scientific community outside the established and controlled workflows and algorithms. To meet the general idea of verification and reproducibility of scientific findings, we offer access to data at the local database upon request at any time. Interested researchers should make their requests to the coordinating PI of the GPS (Achim Fieß; achim.fiess@unimedizin-mainz.de). More detailed contact information is available at the homepages of the UM (www.unimedizin‐mainz.de).
