# Supplementary material for: Breastfeeding in term and preterm infants with and without growth restriction: A 50‐year analysis of incidence and duration
Source: Int J Gynaecol Obstet. 2026 Jan 23;173(3):1503–15. doi: 10.1002/ijgo.70770 (PMC13173627; doi:10.1002/ijgo.70770)
Supplement: Supplementary file 1 — Appendix S1: [file IJGO-173-1503-s001.pdf]

Table of Contents

Supplemental Figure 1 - Distribution of participant age at study examination.

Supplemental Table 1 - Maternal characteristics, neonatal parameters and adult health parameters stratified by breastfeeding groups (no breastfeeding, delayed breastfeeding and immediate breastfeeding.

Supplemental Table 2 - Association analyses of breastfeeding rate after birth of infants born preterm and full-term with continuous parameters (n=1559).

Supplemental Table 3 - Association analyses of breastfeeding initiation, breastfeeding duration after birth of infants born preterm and full-term with continuous parameters (n=1038).

Supplemental Figure 1. Distribution of participant age at study examination.

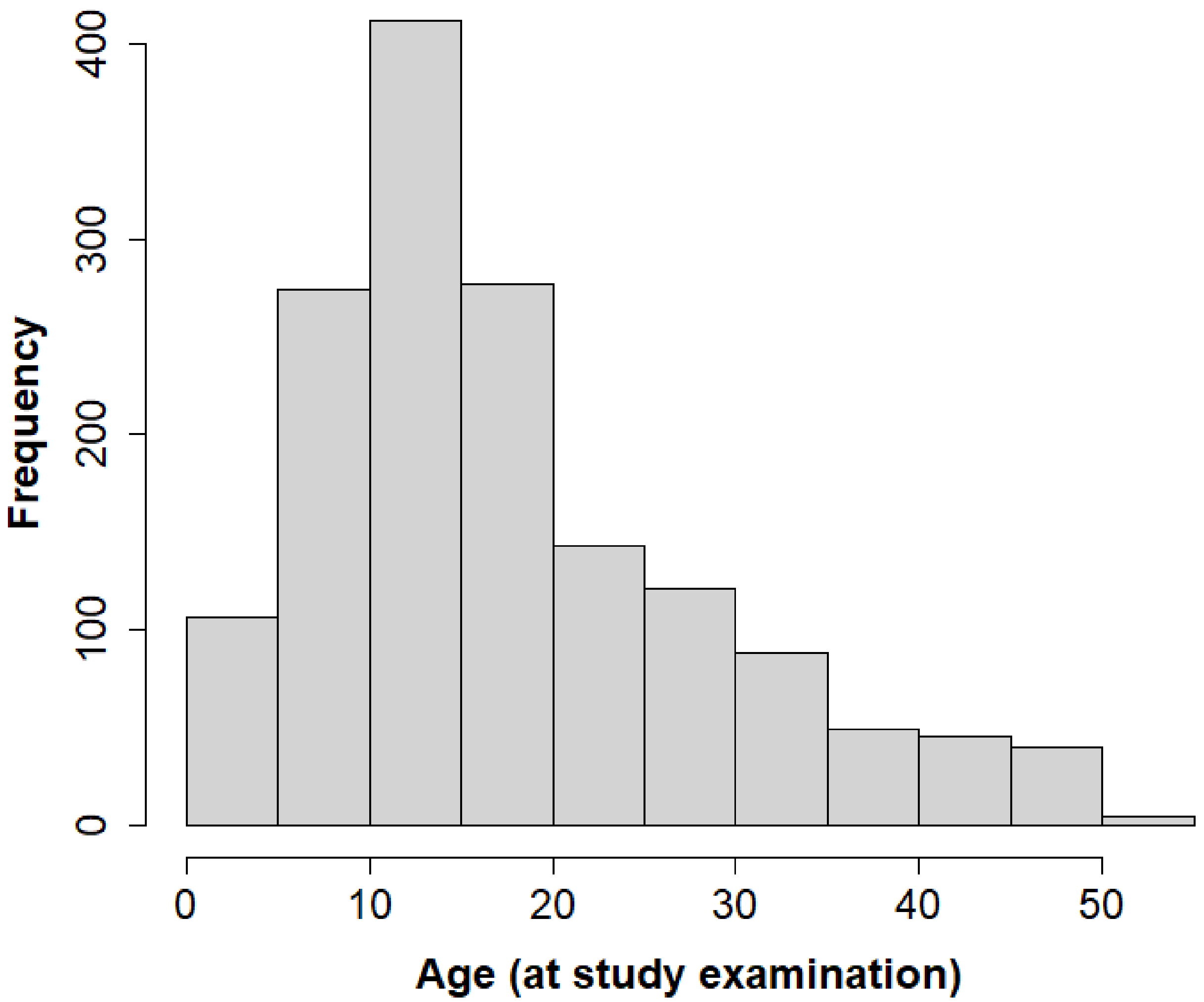

Supplemental Table 1. Maternal characteristics, neonatal parameters and adult health parameters stratified by breastfeeding groups (no breastfeeding, delayed breastfeeding and immediate breastfeeding.

	No Breastfeeding	Delayed Breastfeeding	Immediate Breastfeeding	p-value
		>1day	<=1day	
Number of participants	521	225	597	
**Maternal characteristics**				
Maternal age at the time of questionnaire completion	50·93 (10·84)	48·13 (8·64)	46·56 (9·08)	0·25
Mother's school-leaving qualification				<0·001
• higher secondary school completion	243 (46·6%)	157 (69·8%)	417 (69·8%)	
• basic or intermediate school	278 (53·4%)	68 (30·2%)	180 (30·2%)	
**Neonatal parameters**				
Gestational age (weeks), Median (IQR)	34·00 [30·00, 38·00]	34·00 [31·00, 37·00]	38·00 [33·00, 39·00]	<0·001
Birth weight percentile, Median (IQR)	31·00 [10·00, 60·00]	35·00 [13·00, 56·00]	41·00 [20·00, 69·00]	<0·001
Multiple birth (yes), n (%)	157 (30·1%)	73 (32·4%)	123 (20·6%)	<0·001
Preeclampsia (yes), n (%)	68 (13·1%)	26 (11·6%)	42 (7·0%)	0·003
Placental insufficiency (yes), n (%)	31 (6·0%)	13 (5·8%)	8 (1·3%)	<0·001
HELLP syndrome (yes), n (%)	17 (3·3%)	15 (6·7%)	14 (2·3%)	0·01
Gestational diabetes (yes), n (%)	26 (5·0%)	20 (8·9%)	56 (9·4%)	0·02
Perinatal adverse events (yes), n (%)	70 (13·4%)	11 (4·9%)	28 (4·7%)	<0·001
Necrotizing enterocolitis, n (%)	15 (2·9%)	2 (0·9%)	5 (0·8%)	0·02
Moderate/severe bronchopulmonary dysplasia, n (%)	59 (11·3%)	10 (4·4%)	22 (3·7%)	<0·001
	**No Breastfeeding**	**Delayed Breastfeeding**	**Immediate Breastfeeding**	**p-value**
Intubation, n (%)	151 (29·0%)	45 (20·0%)	76 (12·7%)	<0·001

Intensive care unit stay (days), median (IQR)	2·00 [0·00, 28·00]	2·00 [0·00, 14·00]	0·00 [0·00, 3·00]	<0·001
**Health parameters in adulthood**				
Diabetes mellitus type 1 (yes)	5 (1·0%)	0 (0·0)	1 (0·2%)	0·08
Diabetes mellitus type 2 (yes)	6 (1·2%)	0 (0·0)	0 (0·0%	0·01
Hyperlipidemia (yes)	6 (2·2%)	0 (0·0)	2 (3·2%)	0·47
Autoimmune disease (yes)	3 (0·6%)	4 (1·8%)	7 (1·3%)	0·32
Epilepsy (yes)	18 (3·6%)	6 (2·7%)	1 (0·2%)	<0·001
Hypertension (yes)*	106 (25·2%)	26 (14·1%)	40 (6·7%)	<0·001
Allergies (yes)	194 (37·9%)	88 (39·5%)	206 (35·9%)	0·60

Legend:
*Arterial hypertension was defined by antihypertensive medication use, systolic blood pressure >140 mmHg, diastolic blood pressure > 90 mmHg, or an established diagnosi s

Supplemental Table 2. Association analyses of breastfeeding rate after birth of infants born preterm and full-term with continuous parameters (n=1559).

	Model with continuous parameters: GA, BW percentile	
	OR (CI_{95})	p-value
**Breastfeeding (yes)**		
Weeks of prematurity	0·92 (0·90, 0·95)	<0·001
Age of mother at birth	0·99 (0·98, 1·06)	0·35
Year of birth	1·05 (1·03, 1·06)	<0·001
Mother's school-leaving qualification (high)	1·59 (1·24, 2·04)	<0·001
Multiple births (yes)	0·90 (0·69, 1·19)	0·48
BW percentile	1·01 (1·00, 1·01)	0·002

Supplemental Table 3. Association analyses of breastfeeding initiation, breastfeeding duration after birth of infants born preterm and full-term with continuous parameters (n=1038).

	Model with continuous parameters: GA, BW percentile	
	OR (CI_{95})	p-value
**Delayed initiation of breastfeeding (> 1 day)**		
Weeks of prematurity	1·47 (1·36, 1·60)	<0·001
Age of mother at birth	1·04 (0·99, 1·10)	0·11
Year of birth	0·96 (0·94, 0·99)	<0·001
Mother's school-leaving qualification (high)	0·95 (0·57, 1·60)	0·85
Multiple births (yes)	2·31 (1·18, 4·68)	0·02
BW percentile	0·99 (0·99, 1·00)	0·17
**Total breastfeeding duration (<6 months)**		
Weeks of prematurity	1·00 (0·97, 1·04)	0·97
Age of mother at birth	0·97 (0·94, 1·00)	0·06
Year of birth	0·96 (0·94, 0·98)	<0·001
Mother's school-leaving qualification (high)	0·66 (0·48, 0·91)	0·01
Multiple births (yes)	1·67 (1·15, 2·43)	0·01
BW percentile	0·99 (0·98, 0·99)	0·004
**Feeding problems after discharge**		
Weeks of prematurity	0·99 (0·93, 1·04)	0·58
Age of mother at birth	0·94 (0·89, 0·99)	0·02
Year of birth	1·03 (1·00, 1·05)	0·06
Mother's school-leaving qualification (high)	1·61 (0·96, 2·77)	0·08
Multiple births (yes)	1·05 (0·57, 1·87)	0·88
BW percentile	0·99 (0·98, 1·00)	0·004

Legend: GA - gestational age; BW - birth weight; Mother's school-leaving qualification (high)* was defined as "high" for higher secondary school completion, such as “Gymnasium” or university degree, and "lower" for basic “Hauptschule” or intermediate school “Realschule”; weeks of prematurity was classified as the difference in weeks between the infant’s actual gestational age and full term (40 weeks), indicating the degree of prematurity
